# Structural equation models to infer relationships between energy-related blood metabolites and milk daily energy output in Holstein cows

**DOI:** 10.1093/jas/skae271

**Published:** 2024-09-16

**Authors:** Sara Pegolo, Marco Aurelio Ramirez Mauricio, Enrico Mancin, Diana Giannuzzi, Vittoria Bisutti, Lucio Flavio Macedo Mota, Paolo Ajmone Marsan, Erminio Trevisi, Alessio Cecchinato

**Affiliations:** Department of Agronomy, Food, Natural resources, Animals and Environment (DAFNAE), University of Padova, Legnaro, Padova, Italy; Department of Agronomy, Food, Natural resources, Animals and Environment (DAFNAE), University of Padova, Legnaro, Padova, Italy; Department of Agronomy, Food, Natural resources, Animals and Environment (DAFNAE), University of Padova, Legnaro, Padova, Italy; Department of Agronomy, Food, Natural resources, Animals and Environment (DAFNAE), University of Padova, Legnaro, Padova, Italy; Department of Agronomy, Food, Natural resources, Animals and Environment (DAFNAE), University of Padova, Legnaro, Padova, Italy; Department of Agronomy, Food, Natural resources, Animals and Environment (DAFNAE), University of Padova, Legnaro, Padova, Italy; Department of Animal Science, Food and Nutrition (DIANA), Università Cattolica del Sacro Cuore, Piacenza, Italy; Department of Animal Science, Food and Nutrition (DIANA), Università Cattolica del Sacro Cuore, Piacenza, Italy; Department of Agronomy, Food, Natural resources, Animals and Environment (DAFNAE), University of Padova, Legnaro, Padova, Italy

**Keywords:** Bayesian network, blood traits, energetic metabolism, dairy cows, genetic parameters, structural equation model

## Abstract

During lactation, high-yielding cows experience metabolic disturbances due to milk production. Metabolic monitoring offers valuable insights into how cows manage these challenges throughout the lactation period, making it a topic of considerable interest to breeders. In this study, we used Bayesian networks to uncover potential dependencies among various energy-related blood metabolites, i.e., glucose, urea, beta-hydroxybutyrate (**BHB**), non-esterified fatty acids (**NEFA**), cholesterol (**CHOL**), and daily milk energy output (**dMEO**) in 1,254 Holstein cows. The inferred causal structure was then incorporated into structural equation models (**SEM**) to estimate heritabilities and additive genetic correlations among these phenotypes using both pedigree and genotypes from a 100k chip. Dependencies among traits were determined using the Hill-Climbing algorithm, implemented with the posterior distribution of the residuals obtained from the standard multiple-trait model. These identified relationships were then used to construct the SEM, considering both direct and indirect relationships. The relevant dependencies and path coefficients obtained, expressed in units of measurement variation of 1σ, were as follows: dMEO → CHOL (0.181), dMEO → BHB (−0.149), dMEO → urea (0.038), glucose → BHB (−0.55), glucose → urea (−0.194), CHOL → urea (0.175), BHB → urea (−0.049), and NEFA → urea (−0.097). Heritabilities for traits of concern obtained with SEM ranged from 0.09 to 0.2. Genetic correlations with a minimum 95% probability (*P*) of the posterior mean being >0 for positive means or <0 for negative means include those between dMEO and glucose (−0.583, *P* = 100), dMEO and BHB (0.349, *P* = 99), glucose and CHOL (0.325, *P* = 100), glucose and NEFA (−0.388, *P* = 100), and NEFA and BHB (0.759, *P* = 100). The results of this analysis revealed the existence of recursive relationships among the energy-related blood metabolites and dMEO. Understanding these connections is paramount for establishing effective genetic selection strategies, enhancing production and animal welfare.

## Teaser

This study investigated the genetic and recursive relationships between energy-related metabolites (i.e., glucose, urea, BHB, NEFA, cholesterol) and the daily milk energy output (dMEO). This information should be considered when developing breeding strategies aimed at improving the animals’ response to metabolic distress.

## Introduction

Production diseases related to poor nutrition or management conditions commonly affect high-yielding dairy cows (Overton and Waldron, 2004). During the transition from gestation to lactation, the dairy cow undergoes tremendous metabolic changes (Mezzetti et al., 2021). Conditions of negative energy balance (**NEB**) (fasting, parturition, and lactation) can be associated with excessive fat mobilization and accumulation in liver cells, with a consequent increase in ketogenesis and alterations in liver physiology and morphology (Andjelić et al., 2022). In due course, cows show a low level of milk yield, an increase in concurrent diseases (e.g., mastitis, metritis, and displaced abomasum), and poor reproductive performance (Overton et al., 2017). Even during ongoing lactation, the regulation of metabolic homeostasis varies markedly depending on the stage of lactation (Drackley, 1999). Several studies have highlighted that immune-metabolic shifts may initiate in late lactation and the dry-off period, leading to enduring effects post-calving and at the early stages of the subsequent lactation (Mezzetti et al., 2019; Premi et al., 2021; Giannuzzi et al., 2023a). In this context, metabolic monitoring provides an in-depth insight into how the cows cope with these challenges along the entire lactation period and can be of great interest to breeders.

Some blood biochemical parameters, such as those related to energy metabolism, can reflect the energy status of the cow and can be useful for early detection and prevention of metabolic and nutritional dysfunctions in dairy herds (Ospina et al., 2010; Giannuzzi et al., 2023a, b). Lipid metabolites can be indicators of metabolic distress and adaptation in cows. Non-esterified fatty acids (**NEFA**) are accumulated as triglycerides (**TGA**) in the liver, primarily due to the decreased hepatic synthesis of very low-density lipoproteins (**VLDL**) (Drackley, 1999). However, in liver lipidosis, endogenous synthesis decreases, leading to a reduction in blood glucose, albumin, globulin, total cholesterol (**CHOL**), TGA, and urea concentrations (Giannuzzi et al., 2021). Changes in blood metabolites [e.g., increase in beta-hydroxybutyrate (**BHB**) and decrease in glucose] during an NEB in later stages of lactation were reported even if they differed in their extent compared to the NEB at the onset of lactation (Gross et al., 2011; Bjerre-Harpøth et al., 2012). However, it has never been investigated in previous studies whether this metabolic discomfort is a consequence of animals being overly productive or, conversely, if animals capable of actively managing their metabolic state are the most productive.

Traditional approaches to studying the associations among multiple related phenotypes have typically involved the use of standard multiple-trait models (**MTM**, Henderson, 1984) or their genomic equivalents (e.g., Calus and Veerkamp 2011; Jia and Jannink 2012). Multiple-trait models have been proven valuable in estimating genetic correlations and improving the prediction accuracy of breeding values for traits with low heritability or limited data via joint modeling with one or more genetically correlated, highly heritable traits (Mrode 2014).

However, the covariances in a MTM represent indirect effects or relationships, potentially causing these models to overlook any underlying casual relationships among phenotypes (Varona and González-Recio, 2023). Structural equation models (**SEM**) are multivariate models, which instead capture direct causal effects or relationships. SEM can postulate unidirectional causality (recursive models) from y_1_ into y_2_ (or vice versa), or mutual causality between y_1_ and y_2_ (simultaneous models). They expand upon the concept of path analysis (Wright, 1921) by allowing the simultaneous estimation of multiple relationships and interactions among variables within a single model. In dairy cows, they have been applied to investigate the genetic architecture of both production (de los Campos et al., 2006; Inoue et al., 2016; Pegolo et al., 2021) and functional traits (Heringstad et al., 2009; Pegolo et al., 2020; Martinez-Castillero et al., 2021).

As far as we are aware, there has not been any previous research exploring the potential presence of causal relationships between energy-related blood metabolites and the daily milk energy output (**dMEO**, MJ/d) in dairy cows. In addition, investigating the genetic correlations between energy-related blood metabolites and nutrient-based energy secreted into milk in dairy cattle is paramount for gaining valuable insights into the genetic factors underlying metabolic processes and milk yield (**MY**). The acquired knowledge serves as a compass for selective breeding programs, contributing not only to the overall productivity of dairy herds but also to the dairy cattle’s well-being.

We hypothesized putative dependencies might exist between dMEO, which is an indicator of the cow’s metabolic load during lactation, and energy-related blood metabolites (i.e., glucose, urea, BHB, NEFA) and total CHOL. Therefore, the aims of this study were 1) to estimate genetic parameters for a set of energy-related blood metabolites and dMEO throughout lactation in dairy cows using MTM; 2) to use Bayesian network (**BN**) for inferring putative dependencies among these phenotypes; 3) to leverage the identified structure in SEM for inferring direct recursive relationships among these phenotypes.

## Materials and Methods

The present study was conducted within the LATSAN and BENELAT projects, aimed at developing short- and long-term interventions for improving animal welfare and efficiency, and the quality of dairy cattle production. The research was approved by the Ethical Animal Care and Use Committee (OPBA, Organismo Preposto al Benessere degli Animali) of the Università Cattolica del Sacro Cuore and by the Italian Ministry of Health (protocol number 510/2019-PR of July 19, 2019).

## Field Data and Sample Collection

Individual milk and blood samples were collected from 1,359 Holstein–Friesian lactating cows belonging to 5 different herds located in northern Italy. All animals were housed in free-stall and fed total mixed ration (**TMR**) based on corn silage, sorghum silage, and concentrates. Drinking water was available through automatic water bowls. Cows with clinical signs of disease or under medical treatment were excluded from the trial. More details about animals, feeding, and herds management conditions are reported in Giannuzzi et al. (2022) and Pegolo et al. (2023). The cows included in this study were in parity order ranging from 1 to 4 and in days in milk (**DIM**) ranging from 5 to 598.

Milk samples were collected once during the evening milking from March 2019 to February 2020 (one or more sampling dates per herd depending on its size, 20 herd-date combinations in total). Within 2 h from collection, a 50 mL aliquot added with bronopol was refrigerated at 4 °C and transported to the laboratory of the Veneto Breeders Association (located in Padua, Italy). The analyses of milk composition were conducted within 48 h of collection. Blood samples (9 mL) were collected once from the jugular vein in the morning before TMR distribution and put in vacuum tubes with 150-lithium heparin USP units (Vacumed; FL Medical, Torreglia, Padua, Italy). Blood and milk sampling were carried out on the same day for each cow.

## Phenotypes

### Milk composition

Analyses of milk quality and composition, including protein, casein, fat, and lactose percentages, and urea (mg/100 g) were carried out with an FT6000 Milkoscan infrared analyzer (Foss A/S, Hillerød, Denmark). As an indication of the cow’s metabolic load for lactation, we calculated the dMEO (MJ/d) from the net energy content (**NEL**, MJ/kg) of the milk. The NEL was estimated according to NASEM (2021):


$$\begin{array}{lll} NEL\left(Mcal/kg\right)= & \;0.3887\times fat\;\%+0.2301\times protein\;\%\; & +0.1653\times lactose\;\% \end{array}$$

where 0.3887, 0.2301, and 0.1653 are the individual heat of combustion coefficients for fat, protein, and lactose, respectively. The NEL measures were converted to megajoules per kilogram and then multiplied by the daily MY (kg/d) of each cow to obtain dMEO values expressed in MJ/d.

### Energy-related blood metabolites

Energy-related blood metabolites were determined as detailed in Mezzetti et al. (2019). In short, blood samples were chilled until centrifugation at 3,500 × *g* for 16 min at 6 °C (Hettich Universal 16R Centrifuge), carried out within 2 h of collection. The resulting plasma was then stored at −20 °C for later analysis. Concentrations of glucose (mmol/L), NEFA (mmol/L), BHB (mmol/L), CHOL (mmol/L), and urea (mmol/L) were determined using the ILAB-650 clinical auto-analyzer (Instrumentation Laboratory-Werfen, Bedford, MA).

## Pedigree and genomic data

After data editing, phenotypes from 1,254 cows registered in the pedigree were retained for subsequent analysis. In total, 105 animals sampled for blood and milk were excluded because they have no pedigree information. The pedigree data was provided by the Italian National Association of Holstein, Brown, and Jersey Breeders (ANAFIBJ) and included a total of 12,545 individual-sire-dam entries. The phenotyped cows were genotyped using the Geneseek Genomic Profiler (GGP) Bovine 100k single-nucleotide polymorphisms (**SNP**) Chip assay (NEOGEN, Lincoln, USA). After filtering out non-autosomal regions, a series of genotype quality control measures were applied. These included the removal of autosomal SNP markers with a minor allele frequency of less than 0.05 and those exhibiting significant deviations from Hardy–Weinberg equilibrium (*P* ≤ 10^−5^). Markers and samples with call rates lower than 0.95 were also excluded from the analysis. After quality controls, genotypes from 1,241 cows and 60,582 SNP were used in subsequent analysis.

## MTM analysis

A Bayesian MTM was fitted to infer heritability, genetic and residual (co)variances of the phenotypes (i.e., dMEO, glucose, CHOL, NEFA, BHB, and urea), with the following model:


$$\begin{array}{l} y=Xb+Wg+Zh+e \end{array}$$

Where **y** is the vector of scaled *t* phenotypes (*t* = 6, all phenotypes were scaled with mean 0 and SD 1, to simplify computations due to the different trait unit of measurement); **b** is the vector of the fixed effects, including 1) the cow’s DIM categorized into 30-d classes (classes: 1 to 11 and ≥12) and 2) parity (classes: 1, 2, 3, ≥4), **g** represents the vector of additive genetic effect, **h** is the random herd-date effect (20 levels); **e** is the vector of the residuals; **X**, **W**, and **Z** are the incidence matrices for fixed effects, additive genetic effect, and the random herd-date effect, respectively.

The **g**, **h**, and **e** vectors are assumed to have independent Gaussian distributions **g** ~ N (0, **H** ⊗ **Σ**_g_), **h** ~ N (0, **I** ⊗ **Σ**_p_), and **e** ~ N (0, **I** ⊗ **Σ**_e_), respectively. Where the term ⊗ is the Kronecker product and **Σ**_g_, **Σ**_h_, and **Σ**_e_ were “unstructured” 6 × 6 variance–covariance matrices of genetic, herd-date, and residuals effects, respectively. In addition, ***I*** is an identity matrix and **H** is a genetic relationship matrix, which combines pedigree and genomic information. The inverse of **H** is given by (Aguilar et al., 2010):


$$\begin{array}{l} H^{-1}=A^{-1}+\left[\begin{array}{ccc} 0 & 0\;0 & G^{-1}-A_{22}^{-1} \end{array}\right] \end{array}$$

where **A**^−1^ and $A_{22}^{-1}$ are the inverse of the pedigree kinship matrix, respectively, for all animals and for only genotyped animals. The pedigree kinship matrix ***A*** is computed tracing back the pedigree up to 3 generations (i.e., 4,749 animals in total).

Heritability is defined as $h^{2}=\sigma_{g}^{2}/(\sigma_{g}^{2}+\sigma_{h}^{2}+\sigma_{e}^{2})$, where $\sigma_{g}^{2}$, $\sigma_{h}^{2}$, and $\sigma_{e}^{2}$ are the additive genetic, herd-date, and residual variances, respectively. The genetic correlations (*r*_g_) between trait pairs are calculated as $\;r_{g}=\sigma_{g_{i}g_{j}}/\sqrt{\left(\sigma_{g_{i}}^{2}\times \sigma_{g_{j}}^{2}\right)}$, where *r*_g_ is genetic correlations, $\sigma_{g_{i}g_{j}}$ is additive genetic covariance between traits *i* and *j*, $\sigma_{g_{i}\;\;\;}^{2}$ is additive genetic variance for trait *i*, and $\sigma_{g_{j}}^{2}$ is additive genetic variance for trait *j*. The residual correlations (*r*_e_) are calculated as $\;r_{e}=\sigma_{e_{i}e_{j}}/\sqrt{\left(\sigma_{e_{i}}^{2}\times \sigma_{e_{j}}^{2}\right)}$, where, *r*_e_ is the residual correlations, $\sigma_{e_{i}e_{j}}$ was residual covariance between trait *i* and *j*, $\sigma_{e_{i}\;\;\;}^{2}$ was residual variance for trait *i*, and $\sigma_{g_{j}}^{2}$ was residual variance for trait *j*.

The marginal posterior distributions were derived using a Markov chain Monte Carlo methodology with Gibbs sampling, using GIBBSF90 + program (Misztal et al., 2018). A total of 500,000 chains were generated, with the initial 50,000 chains discarded as burn-in, and subsampling was performed every 100 samples for subsequent analysis, based on visually inspecting convergence from the posterior distributions. Posterior means and the lower and upper bounds of the highest 95% posterior density intervals (**HPD95**%) were calculated for all model parameters using the POSTGIBBSF90 program (Misztal et al., 2018).

For the correlations, in addition to the means of each marginal posterior distribution, we also estimated the probability of each mean being greater than 0 when the mean was positive or lower than 0 when the mean was negative (*P*). All estimates with *P* greater than 95% (*P*) were considered “relevant” correlations.

## Bayesian network algorithm

Bayesian network structure learning algorithms were applied to the vector of residuals from MTM for inferring putative dependencies among traits using the BN, which is a graphical model where the nodes are the phenotypes and the edges are conditional dependencies among them. For this analysis, we selected the Hill-Climbing (**HC**) algorithm, implemented through the R package *bnlearn* (Scutari, 2010). The HC algorithm employs a score-based approach, evaluating candidates in the Bayesian network to maximize the Bayesian Information Criterion (**BIC**) scoring function using heuristic search algorithms (Adhitama and Saputro, 2022). To estimate the edge strength and uncertainty of direction, we applied a bootstrap procedure with 50,000 samples. Since the edge strength refers to the degree of association or dependency between 2 variables represented by an edge in the network, we applied a strength threshold of 95% to keep only high-confidence relationships (Scutari and Nagarajan, 2013).

## Structural equation model

Structural equation models were employed to estimate the causal coefficients based on the network structure obtained from the Bayesian network algorithm. This approach, integrating a SEM framework with a causal structure and random additive genetic effects, was proposed by Gianola and Sorensen (2004), and adapted according to Mora et al. (2022) as follows:


$$\begin{array}{l} y=\left(\lambda ⊗I_{n}\right)y+Xb+Wa+Zh+e\; \end{array}$$

In the SEM, we included the same effects as in the MTM, with the addition of the covariate effects of each phenotype on the others based on the Bayesian network structure. The matrix **Λ** contained all the coefficients for these covariate effects; when no effect was assumed, a zero was assigned. Thus, **Λ** serves as a sort of “incidence matrix” that describes the recursive relationships among traits. In essence, these terms encapsulated the potential effect of the *i*th trait on the *j*th trait at the phenotypic level. Specifically, in our case, **Λ** was a 6 × 6 matrix of structural coefficients inferred using a Bayesian algorithm. The matrix **Λ** was defined as follows:


$$\begin{array}{l} \lambda =\left[\begin{array}{ccccccccccccccccccccccccccccccc} 0 & 0 & 0 & 0 & 0 & 0\;0 & 0 & 0 & 0 & 0 & 0\;\lambda_{dMEO\to CHOL} & 0 & 0 & 0 & 0 & 0\;0 & 0 & 0 & 0 & 0 & 0\;\lambda_{dMEO\to BHB} & \lambda_{glucose\to BHB} & 0 & 0 & 0 & 0\;\lambda_{dMEO\to urea} & \lambda_{glucose\to urea} & \lambda_{CHOL\to urea} & \lambda_{NEFA\to urea} & \lambda_{BHB\to urea} & 0 \end{array}\right] \end{array}$$

where each $\;\;\;\lambda_{i\to j}$ is a structural coeﬃcient describing the magnitude of the causal eﬀect of the *i* phenotype on the *j* phenotype.

The paths obtained from the algorithm are $\lambda_{dMEO\;\to \;CHOL}$: dMEO affecting CHOL; $\lambda_{dMEO\;\to \;BHB}$: dMEO affecting BHB, $\lambda_{dMEO\;\to \;urea}$: dMEO affecting urea, $\lambda_{glucose\;\to \;BHB}$: glucose affecting BHB, $\lambda_{glucose\;\to \;urea}$: glucose affecting urea, $\lambda_{CHOL\;\to \;urea}$: CHOL affecting urea, $\lambda_{NEFA\;\to \;urea}$: NEFA affecting urea and $\lambda_{BHB\;\to \;urea}$: BHB affecting urea, following the nomenclature suggested by López de Maturana et al. (2007).

In SEM, it is noteworthy that the “unstructured” residual covariance matrix found in MTM was substituted with the identity matrix to ensure identifiability, according to Varona et al. (2007) and Valente et al. (2013). This alteration implies that the phenotypes possess independent residuals. A Bayesian approach was applied to fit SEM using GIBBSF90 + (Misztal et al., 2018). The posterior means and HPD95 were calculated for the structural coefficients using the POSTGIBBSF90 program (Misztal et al., 2018) as detailed for the MTM analysis. In SEM, heritability and genetic correlation were calculated using the same equation as in the MTM. Residual correlation was not calculated in SEM, as residuals were independent among traits.

## Results and Discussion

### Multi-trait genetic correlations

Descriptive statistics of milk production traits and energy-related blood metabolites are reported in Table 1. Cows enrolled in this study had an average milk production of 33.39 ± 9.26 kg/d with an average milk composition of 3.43 ± 0.34 protein percentage, 3.70 ± 0.89 fat percentage, and 4.86 ± 0.25 lactose percentage. Average milk energy production levels were 99.81 ± 26.23 MJ/d. With respects to energy-related blood metabolites, glucose was 4.21 ± 0.45 mmol/L, CHOL was 5.27 ± 1.26 mmol/L, NEFA 0.13 ± 0.16 mmol/L, BHB 0.55 ± 0.21 mmol/L, urea 6.12 ± 1.21 mmol/L. Among energy-related metabolites, NEFA displayed the largest variation. Animals were in the range of physiological values for all metabolites except in the case of blood urea, which for 219 animals had values > 7.17 mmol/L exceeding the physiological threshold of 6.78 mmol/L (Premi et al., 2021). This can be explained by the fact that animals enrolled in this study were high-producing cows with a high level of protein content in their diet (16% on average; Giannuzzi et al., 2022) and likely a very high feed intake.

**Table 1.** Descriptive statistics for test-day milk production, composition and blood traits related to energy metabolism in Holstein cows

Traits^1^	*N*	Mean	SD	P1	P99
Milk traits					
Milk yield, kg/d	1,254	33.39	9.26	12.07	56.39
Protein, %	1,254	3.43	0.34	2.69	4.37
Fat, %	1,254	3.7	0.89	1.56	6.12
Lactose, %	1,254	4.86	0.25	4	5.29
dMEO, MJ/d	1,252	99.81	26.23	38.56	160.24
Energy-related blood metabolites					
BHB, mmol/L	1,254	0.55	0.21	0.27	1.28
CHOL, mmol/L	1,253	5.27	1.26	2.23	8.32
Glucose, mmol/L	1,254	4.21	0.45	2.95	5.08
NEFA, mmol/L	1,253	0.13	0.16	0.03	0.69
Urea, mmol/L	1,254	6.12	1.21	3.23	8.91

^1^BHB, beta-hydroxybutyrate; CHOL, cholesterol; dMEO, daily milk energy output; MY, milk yield; NEFA, non-esterified fatty acids; P1, 1st percentile; P99, 99th percentile.

**Figure 1.**
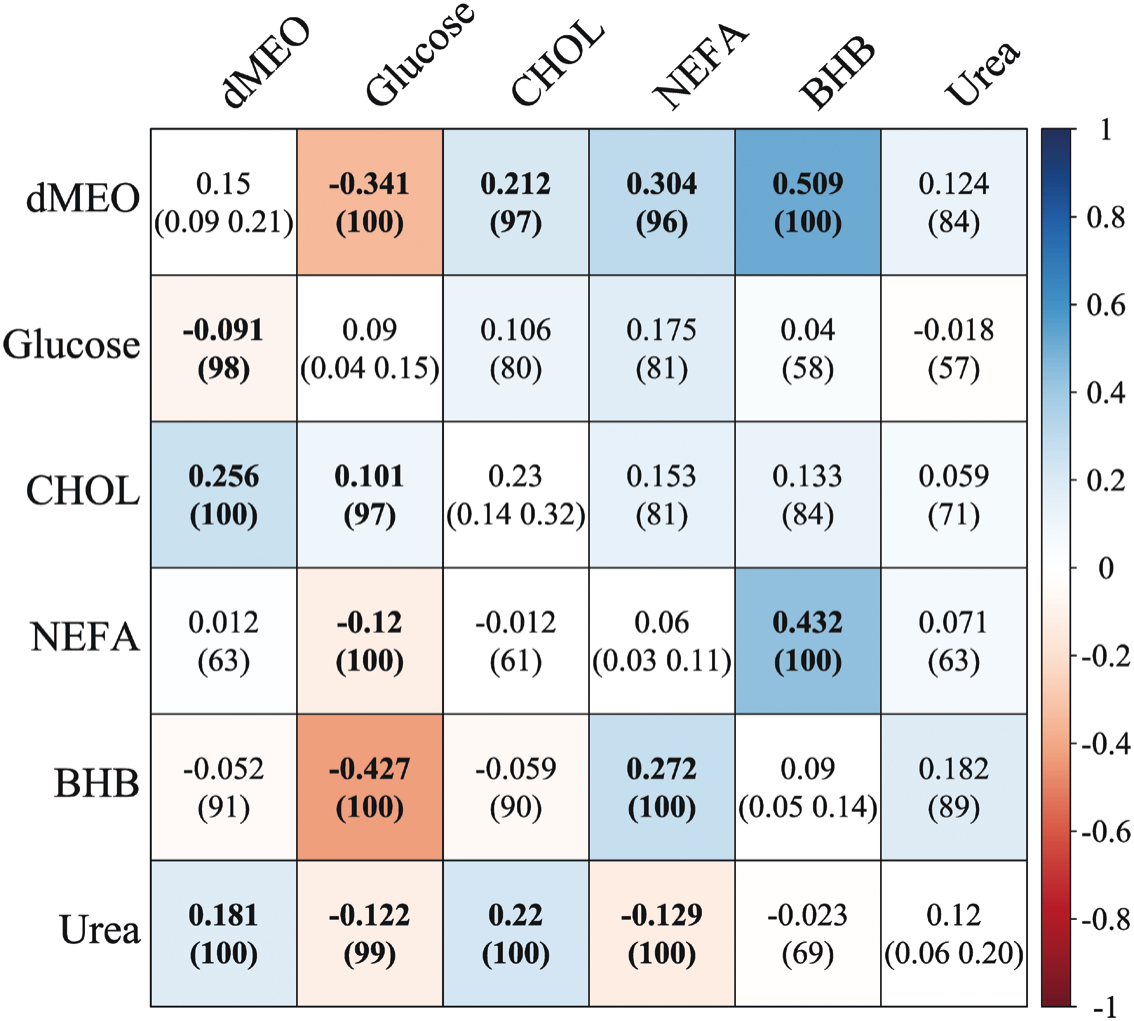
Genetic (upper triangular) and residual (lower triangular) correlations, and heritabilities (diagonal) estimates for the investigated traits obtained with the multiple-trait model (MTM). The probability of being above 0 for positive estimates, and below 0 for negative estimates for the genetic correlations, and the highest 95% posterior density intervals (HPD95%) from the estimated marginal densities for the heritabilities are given in parentheses. Boldface indicates equal or greater 95% of posterior probability accumulated above 0 (positive estimates) or below 0 (negative estimates) for the correlations. BHB, beta-hydroxybutyrate; CHOL, cholesterol; dMEO, daily milk energy output; NEFA, non-esterified fatty acids.

Posterior estimates of the genetic and residual correlations together with heritability estimates obtained for the investigated traits with the MTM are reported in Figure 1. Heritability estimates were low (<0.10) for glucose, NEFA and BHB, low-moderate (<0.20) for urea and dMEO, and moderate for CHOL (0.23; HPD95% = 0.14, 0.32). Heritability values for blood BHB and NEFA were in line with previous results obtained in early lactation (Belay et al., 2017; Benedet et al., 2020; Mehtiö et al., 2020) as well as across the whole lactation (Lou et al., 2022). The literature reveals slightly greater heritability values for blood glucose compared to our findings, as indicated by studies such as Ahn et al. (2006) in cattle (0.21 ± 0.15) and Hayhurst et al. (2009) in calves (heritability range in 3 datasets: 0.23 ± 0.11; 0.13 ± 0.06; 0.20 ± 0.10). The low heritability values obtained for glucose, NEFA and BHB highlight that these traits are influenced more by management practices and/or environmental factors than by the genetic factors of the animals. The literature, especially studies such as those conducted by Kato et al. (2011) for blood CHOL and by Van Den Berg et al. (2021) for blood urea, reveals heritability values comparable to our findings. This supports the concept that these traits have a more pronounced genetic basis. Heritability values for dMEO are in line with previous evidence on a larger database (Martinez-Castillero et al., 2020), and they appear to mirror the genetic inheritance patterns observed for MY, as evidenced by studies such as in Mota et al. (2020).

Relevant low-moderate positive genetic correlations were found between dMEO and CHOL (0.212; *P* = 97), between dMEO and NEFA (0.304; *P* = 96), and moderate positive between dMEO and BHB (0.509, *P* = 100) and between NEFA and BHB (0.432, *P* = 100). The positive genetic relationship between dMEO and CHOL suggests that there may be a shared genetic influence or common genetic factors underlying both traits. Despite the extensive knowledge of other species, CHOL metabolism in ruminants is still not clear. Huge differences in milk CHOL concentration are observed between breeds, managing strategies, subclinical disease conditions, individuals, and lactating period, but the genetic factors working in the control of CHOL homeostasis have not been fully understood. The mammary gland has demonstrated a capacity for CHOL production, yet the CHOL synthesized in the mammary gland may constitute only around 20% of the total (Viturro et al., 2009). Most of the milk CHOL content is produced in the liver and then transported through the bloodstream to reach its destination. Because energy requirements vary during different lactation stages, fluctuations in blood and milk CHOL may be a result of metabolic adaptations in response to the energy demands specific to each lactation stage (Gross et al., 2015). Moreover, some research indicates that changes in the expression of certain genes may contribute to this variability. These include genes that regulate CHOL synthesis (HMGCS1, HMGCR, and FDFT) and genes involved in lipid metabolism (SREBP1 and 2) (Viturro et al., 2009; Kessler et al., 2014). In addition, previous studies observed that subclinical disease conditions are associated with a reduction in the circulating lipoproteins and that low levels of CHOL are maintained in animals which experienced inflammatory conditions after the acute phase (Bertoni et al., 2008; Trevisi et al., 2012).

The positive genetic correlations between dMEO and BHB and between dMEO and NEFA were in line with previous results (Belay et al., 2017; Cecchinato et al., 2018; Benedet et al., 2020), indicating that the best individuals for milk energy output were those with offspring exhibiting on average greater blood BHB and NEFA. These results might be explained by the fact that we excluded animals with clinical conditions, and therefore, in strong NEB. Nutritional and metabolic status are strictly related to blood levels of NEFA and BHB. The main blood indicators of lipomobilization in ruminants are BHB, the most important and abundant ketone body, and NEFA (Djoković et al., 2017). Preferably, NEFA are accumulated as TGA in the liver, primarily because of a decrease in the VLDL synthesis by hepatocytes (Andjelić et al., 2022). The bovine liver has indeed a limited capacity to metabolize NEFA into TAG: they can be oxidized or exported as VLDL. When the limit is reached, TAG accumulates in the liver. Acetyl CoA, a byproduct of fatty acid oxidation, not utilized in the tricarboxylic acid cycle, is converted into ketone bodies such as acetone, acetoacetate, and BHB. These may appear in the blood, milk, and urine. High-producing cows undergo homeorhesis, directing all metabolic pathways toward milk synthesis in the mammary gland (Costa et al., 2019). Consequently, as the energy requirements for milk synthesis increase, circulating blood NEFA and ketone bodies also increase due to fat reserve mobilization (Carvalho et al., 2019). In line with these physiological mechanisms, we found a low-moderate negative genetic correlation between dMEO and glucose (−0.341; *P* = 100). Due to insufficient feed intake, a shift occurs in substrate availability for hepatic gluconeogenesis (i.e., fractional lactate, amino acids [especially alanine]), and glycerol utilization increases, but fractional propionate utilization for gluconeogenesis decreases (Reynolds et al., 2003; Aschenbach et al., 2010). These changes in substrate availability might favor gene expression of some hepatic enzymes involved in gluconeogenesis with different time patterns as lactation proceeds (Weber et al., 2013). Interestingly, pyruvate carboxylase mRNA abundance increased during feed restriction in dairy cows (Velez and Donkin, 2005), indicating comparable mRNA expression patterns of pyruvate carboxylase during the early transition period and feed restriction. Our results might suggest that cows with greater genetic merit for milk energy output might have a reduced plasticity in energy partitioning with respect to glucose metabolism with consequent lower adaptability to feed restriction.

### Network structure and structural equation modeling

**Figure 2.**
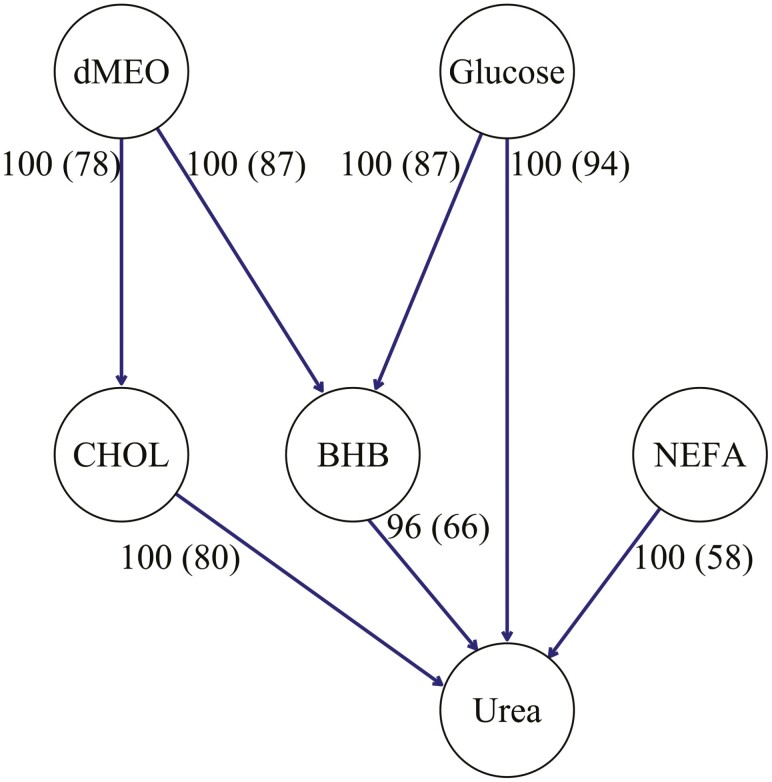
Bayesian network (BN) structure inferred from the vector of the residuals using the HC algorithm. Structure learning test was performed with 50,000 bootstrap samples. The percentages reported beside edges indicate the proportion of bootstrap samples supporting the edge and (in parentheses) the proportion having the direction shown. BHB, beta-hydroxybutyrate; CHOL, cholesterol; dMEO, daily milk energy output; NEFA, non-esterified fatty acids.

Bayesian networks serve as models that depict how random variables, such as traits, are interrelated through conditional dependencies. Bayesian networks have been applied in the context of animal breeding to investigate several traits simultaneously, with respect to their genetic and residual relationships, for generating hypotheses regarding the causal nature of the identified connections among phenotypes (de los Campos et al., 2006; Bouwman et al., 2014; Pegolo et al., 2020). Two primary types of algorithms are used for learning BN: constraint-based and score-based algorithms. The former method employs a series of tests for conditional independence to understand the network among variables, while the latter assesses the fit of numerous (preferably all) potential networks to empirical data, utilizing a scoring system. Herein, the score-based BN algorithm HC was applied to the vector of residuals from the MTM analysis to identify putative dependencies among phenotypes free of “genetic confounders”. The results obtained are displayed in Fig. 2. All the relationships among the investigated traits were identified with high confidence (>95% edge strength). In this network, we found direct dependences between dMEO and CHOL (78% of bootstrap samples), between dMEO and BHB (87% of bootstrap samples), between glucose and BHB (87% of bootstrap samples), between glucose and urea (94% of bootstrap samples), and between NEFA and urea (58% of bootstrap samples). The paths between dMEO and urea are mediated by CHOL and by BHB. Besides the direct dependency between glucose and urea, an indirect path mediated by BHB also occurred. Direct connections were also found between CHOL and urea (80% of bootstrap samples) and between BHB and urea (66% of bootstrap samples). Then, we modeled the derived BN structure for the examined traits using a set of SEM equations. This allowed us to infer structural coefficients and genetic correlations for the traits under investigation. The inferred path coefficients (λ) are reported in Table 2. All the coefficients were relevant except for the path BHB → urea. The paths dMEO → CHOL, CHOL → urea had positive coefficients, while dMEO → BHB, glucose → BHB, glucose → urea and NEFA → urea had negative coefficients. The largest structural coefficients were found for the path glucose → BHB (−0.550; HPD95% = −0.68, −0.44) and the path glucose → urea (−0.194; HPD95% = −0.31, −0.07) while NEFA → urea had the lowest one (−0.097; HPD95% = −0.16, −0.04). Translating the paths glucose → BHB and glucose → urea into units of measurement for the traits, the lambda values signify that a variation of 1σ in glucose (0.45 mmol/L) leads to a corresponding variation of −0.12 mmol/l in BHB and −0.23 mmol/L in urea. These relationships should be interpreted as phenotypic dependencies, as they arise from residuals devoid of genetic effects. Glucose blood concentration is a direct indicator of energy balance in the organism. Elevated blood glucose levels, stemming from an appropriate energy level in the diet, indicate a reduced need for the animal to derive energy from other sources, such as ketone bodies (Guliński, 2021). Instead, it is expected an adequate energy supply for rumen microbial protein synthesis and animals’ metabolic synthesis (Wang et al., 2021). Specifically, it has been proved that the increase in dietary energy levels promotes rumen energy productivity and rumen microbial protein yield by improving levels of electron transport phosphorylation and substrate-level phosphorylation coupled to glucose fermentation in the ruminal microbiome. This, in turn, leads to a decreased amount of ammonia to be excreted through urea (Lu et al., 2019). It is, however, crucial to consider that all the animals in the study fell within the range of physiological values, except for blood urea, influenced by the high-protein content of their diet and/or the feed intake. Consequently, the magnitude of the coefficients may vary when pathological animals are included.

**Table 2.** Posterior means and 95 % highest posterior density intervals of structural coefficients (λ)^1^

Path^2^	Structural coefficient (λ)^3^	HPD95%^4^	
dMEO → BHB	−0.149	−0.24	−0.06
dMEO → CHOL	0.181	0.1	0.26
dMEO → urea	0.038	−0.05	0.13
Glucose → BHB	−0.55	−0.68	−0.44
Glucose → urea	−0.194	−0.31	−0.07
CHOL → urea	0.175	0.06	0.29
NEFA → urea	−0.097	−0.16	−0.04
BHB → urea	−0.049	−0.13	0.03

^1^Obtained with structural equation modeling based on the network structure inferred with the Hill-Climbing algorithm.

^2^BHB, beta-hydroxybutyrate; CHOL, cholesterol; dMEO, daily milk energy output; NEFA, non-esterified fatty acids.

^3^1-unit (SD) of increase in the upstream trait is associated with a λ unit (SD) change in the affected trait.

^4^HPD95%, highest 95% potserior density intervals.

**Figure 3.**
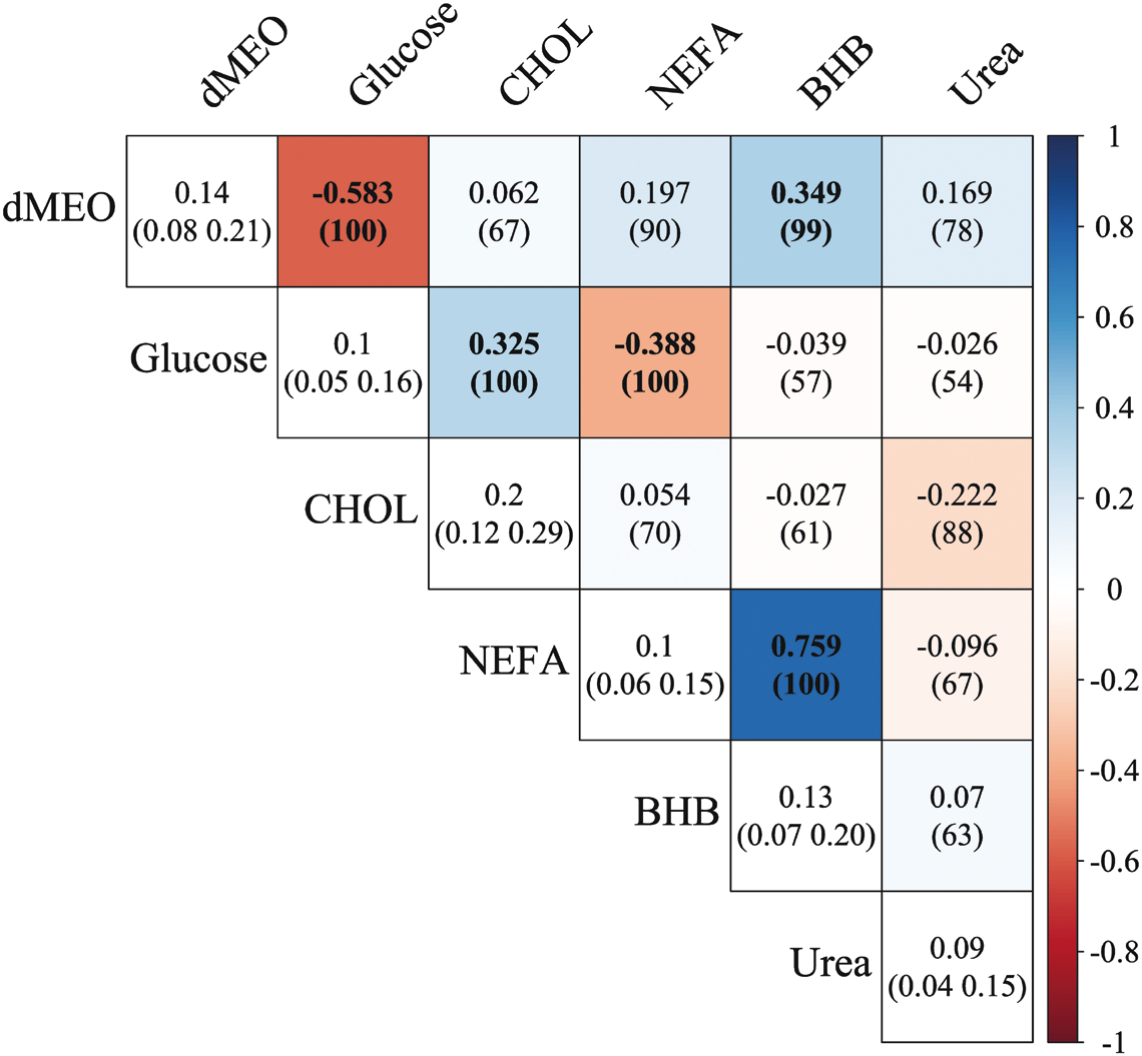
Genetic correlations and heritabilities (diagonal) estimates for the investigated traits using the structure equation model (SEM). The probability of being above 0 for positive estimates, and below 0 for negative estimates for the correlations, and the highest 95% posterior density intervals (HPD95%) from the estimated marginal densities for the heritabilities are given in parenthesis. Boldface indicates equal or greater 95% posterior probability accumulated above 0 (positive estimates) or below 0 (negative estimates) for the genetic correlations. BHB, beta-hydroxybutyrate; CHOL, cholesterol; dMEO, daily milk energy output; NEFA, non-esterified fatty acids.

The results of heritabilities and genetic correlations obtained including the causal structures identified using BN are displayed in Figure 3. Heritability was low for blood urea (≤0.10), glucose and NEFA, and low-moderate (≤0.20) for dMEO, BHB, and CHOL. Moderate to strong negative genetic correlations were found between dMEO and glucose (−0.583), between glucose and NEFA (−0.388) and strong positive between NEFA and BHB (0.759).

The metabolite concentrations in high- and low-genetic merit cows often align with expectations for animals with poorer estimated breeding values for milk yield, featuring greater NEFA, elevated β-hydroxybutyrate, and lower glucose levels (Veerkamp et al., 2003). However, the differences in metabolites are not consistently evident. One contributing factor could be the absence of discernible variations in energy balance among genetic groups, particularly noted in smaller studies such as those by Lucy and Crooker (2001). Additionally, the interplay between genetic merit and diet could influence metabolite concentrations, as demonstrated in the case of glucose by Davey et al. (1983) and Flux et al. (1984). It is worth mentioning, however, that in these evaluations, information about the animals’ feed intake is often missing. In healthy animals, indeed, no metabolic differences are expected in the case of nutrient balance. For a better assessment, it would be crucial to acquire information on individual animals’ feed intake and/or energy and protein balance.

Valente et al. (2013) reported that no information is lost in standard settings by using MTM-based predictions, even if traits are causally associated. In SEM, genetic effects are depicted as directly influencing each trait, independently of any mediation by other traits within the model. Conversely, in MTM, genetic effects are portrayed as encompassing overall influences on each trait, mediating direct and indirect genetic effects that are differentiated in SEM. In breeding programs, however, the focus typically revolves around overall genetic effects. On the other hand, by explicitly modeling the interconnections among phenotypes, SEM can provide insights into the underlying biological processes driving trait variation and a more accurate prediction of intervention outcomes.

Within the complex landscape of dairy cattle breeding, determining the optimal selection direction for metabolic traits in dairy cattle poses a significant challenge. Specifically, a pivotal decision revolves around determining the merit of either increasing or decreasing blood levels of these indicators. Veerkamp et al. (2003) suggested that the selection for yield has altered energy partitioning rules, influencing the growth hormone/insulin-like growth factor 1 axis. This is supported by the observation that NEFA and BHB concentrations, as well as growth hormone secretion, are generally elevated in high genetic merit cows. In contrast, glucose levels in these cows tend to be lower. The challenge arises from the delicate balance required for maintaining both metabolic efficiency and milk production. Elevated blood levels of energy metabolites may signify a high metabolic load, ensuring a healthy and productive state, but an excess might lead to metabolic disorders. On the other hand, lowering these indicators and silencing their signals could be pursued to reduce health impairment but may inadvertently compromise the overall physiology of the animals. Hence, striking the right balance necessitates a better understanding of the genetic interplay and its implications for both metabolic processes and MY. When considering genetic selection for blood metabolic traits in dairy cattle, it might be hypothesized that targeting an intermediate-optimal range of animals, rather than those at the extreme ends, could be the most effective strategy.

## Conclusion

In conclusion, the HC algorithm suggested the existence of causal relationships between the studied traits. Therefore, understanding the causal links between energy-related blood metabolites and milk dMEO could prove strategic when designing breeding programs because the effect of external interventions can be better predicted. The causal structure can give more insight into underlying energy metabolism mechanisms and SEM can predict conditional changes due to such interventions.

## Abbreviations

BHB: beta-hydroxybutyrate
BIC: Bayesian Information Criterion
BN: Bayesian network
CHOL: cholesterol
dMEO: daily milk energy output
DIM: days in milk
HC: Hill-Climbing
HPD95%: highest 95% posterior density intervals
MAF: minor allele frequency
MY: milk yield
MTM: multiple-trait models
NEL: net energy content
NEB: negative energy balance
NEFA: non-esterified fatty acids
SEM: structural equation models
SNP: single-nucleotide polymorphisms
TCA: tricarboxylic acid cycle
TGA: triglycerides
TMR: total mixed ration
VLDL: very low-density lipoproteins

## References

[CIT0001] Adhitama, R. P., and D. R. S. Saputro. 2022. Hill climbing algorithm for Bayesian network structure. AIP Conf. Proc. 2511:020035. doi:10.1063/5.0099793

[CIT0002] Aguilar, I., I. Misztal, D. L. Johnson, A. Legarra, S. Tsuruta, and T. J. Lawlor. 2010. Hot topic: a unified approach to utilize phenotypic, full pedigree, and genomic information for genetic evaluation of Holstein final score. J. Dairy Sci. 93:743–752. doi:10.3168/jds.2009-2730 20105546

[CIT0003] Ahn, B. S., B. S. Jeon, E. G. Kwon, M. A. Khan, H. S. Kim, J. C. Ju, and N. S. Kim. 2006. Estimation of genetic parameters for daily milk yield, somatic cell score, milk urea nitrogen, blood glucose and immunoglobulin in Holsteins. Asian-Aust. J. Anim. Sci. 19:1252–1256. doi:10.5713/ajas.2006.1252

[CIT0004] Andjelić, B., R. Djoković, M. Cincović, S. Bogosavljević-Bošković, M. Petrović, J. Mladenović, and A. Čukić. 2022. Relationships between milk and blood biochemical parameters and metabolic status in dairy cows during lactation. Metabolites. 12:733. doi:10.3390/metabo12080733 36005606 PMC9412388

[CIT0005] Aschenbach, J. R., N. B. Kristensen, S. S. Donkin, H. M. Hammon, and G. B. Penner. 2010. Gluconeogenesis in dairy cows: the secret of making sweet milk from sour dough. IUBMB Life 62:869–877. doi:10.1002/iub.400 21171012

[CIT0006] Belay, T. K., B. S. Dagnachew, Z. M. Kowalski, and T. Ådnøy. 2017. An attempt at predicting blood β-hydroxybutyrate from Fourier-transform mid-infrared spectra of milk using multivariate mixed models in Polish dairy cattle. J. Dairy Sci. 100:6312–6326. doi:10.3168/jds.2016-12252 28571989

[CIT0007] Benedet, A., A. Costa, M. De Marchi, and M. Penasa. 2020. Heritability estimates of predicted blood β-hydroxybutyrate and nonesterified fatty acids and relationships with milk traits in early-lactation Holstein cows. J. Dairy Sci. 103:6354–6363. doi:10.3168/jds.2019-17916 32359995

[CIT0008] Bertoni, G., E. Trevisi, X. Han, and M. Bionaz. 2008. Effects of inflammatory conditions on liver activity in the puerperium and consequences for performance in dairy cows. J. Dairy Sci. 91:3300–3310. doi:10.3168/jds.2008-0995 18765589

[CIT0009] Bjerre-Harpøth, V., N. C. Friggens, V. M. Thorup, T. Larsen, B. M. Damgaard, K. L. Ingvartsen, and K. M. Moyes. 2012. Metabolic and production profiles of dairy cows in response to decreased nutrient density to increase physiological imbalance at different stages of lactation. J. Dairy Sci. 95:2362–2380. doi:10.3168/jds.2011-4419 22541465

[CIT0010] Bouwman, A. C., B. D. Valente, L. L. G. Janss, H. Bovenhuis, and G. J. M. Rosa. 2014. Exploring causal networks of bovine milk fatty acids in a multivariate mixed model context. Genet. Sel. Evol. 46:2. doi:10.1186/1297-9686-46-2 24438068 PMC3922748

[CIT0011] Calus, M. P., and R. F. Veerkamp. 2011. Accuracy of multi-trait genomic selection using different methods. Genet. Sel. Evol. 43:26. doi:10.1186/1297-9686-43-26. 21729282 PMC3146811

[CIT0012] Carvalho, M. R., F. Peñagaricano, J. E. P. Santos, T. J. DeVries, B. W. McBride, and E. S. Ribeiro. 2019. Long-term effects of postpartum clinical disease on milk production, reproduction, and culling of dairy cows. J. Dairy Sci. 102:11701–11717. doi:10.3168/jds.2019-17025 31548073

[CIT0013] Cecchinato, A., T. Bobbo, P. L. Ruegg, L. Gallo, G. Bittante, and S. Pegolo. 2018. Genetic variation in serum protein pattern and blood β-hydroxybutyrate and their relationships with udder health traits, protein profile, and cheese-making properties in Holstein cows. J. Dairy Sci. 101:11108–11119. doi:10.3168/jds.2018-14907 30316608

[CIT0014] Costa, A., N. Lopez-Villalobos, N. W. Sneddon, L. Shalloo, M. Franzoi, M. De Marchi, and M. Penasa. 2019. Invited review: milk lactose-current status and future challenges in dairy cattle. J. Dairy Sci. 102:5883–5898. doi:10.3168/jds.2018-15955 31079905

[CIT0015] Davey, A. W. F., C. Grainger, D. D. S. MacKenzie, D. S. Flux, G. F. Wilson, I. M. Brookes, and C. W. Holmes. 1983. Nutritional and physiological studies of differences between Friesian cows of high and low genetic merit. Proc. New Zealand Soc. Anim. Prod. 43:67–70.

[CIT0016] De los Campos, G., D. Gianola, and B. Heringstad. 2006. A structural equation model for describing relationships between somatic cell score and milk yield in first-lactation dairy cows. J. Dairy Sci. 89:4445–4455. doi:10.3168/jds.S0022-0302(06)72493-6 17033034

[CIT0017] Djoković, R., V. Kurćubić, Z. Ilić, M. Cincović, M. Lalović, B. Jašović, and J. Bojkovski. 2017. Correlation between blood biochemical metabolites milk yield, dry matter intake and energy balance in dairy cows during early and mid lactation. Adv. Diabetes Metab. 5:26–30. doi:10.13189/adm.2017.050202

[CIT0018] Drackley, J. K. 1999. ADSA Foundation Scholar Award. Biology of dairy cows during the transition period: the final frontier? J. Dairy Sci. 82:2259–2273. doi:10.3168/jds.s0022-0302(99)75474-3 10575597

[CIT0019] Flux, D. S., D. D. S. Mackenzie, and G. F. Wilson. 1984. Plasma metabolite and hormone concentrations in Friesian cows of differing genetic merit measured at two feeding levels. Anim. Prod. 38:377–384. doi:10.1017/s0003356100041568

[CIT0020] Giannuzzi, D., R. Tessari, S. Pegolo, E. Fiore, M. Gianesella, E. Trevisi, P. A. Marsan, M. Premi, F. Piccioli-Cappelli, F. Tagliapietra, et al. 2021. Associations between ultrasound measurements and hematochemical parameters for the assessment of liver metabolic status in Holstein-Friesian cows. Sci. Rep. 11:1–15. doi:10.1038/s41598-021-95538-x 34381105 PMC8357813

[CIT0021] Giannuzzi, D., A. Toscano, S. Pegolo, L. Gallo, F. Tagliapietra, M. Mele, A. Minuti, E. Trevisi, P. A. Marsan, S. Schiavon, et al. 2022. Associations between milk fatty acid profile and body condition score, ultrasound hepatic measurements and blood metabolites in Holstein cows. Animals. 12:1–23. doi:10.3390/ani12091202 PMC9104722 35565628

[CIT0022] Giannuzzi, D., L. F. M. Mota, S. Pegolo, F. Tagliapietra, S. Schiavon, L. Gallo, P. A. Marsan, E. Trevisi, and A. Cecchinato. 2023a. Prediction of detailed blood metabolic profile using milk infrared spectra and machine learning methods in dairy cattle. J. Dairy Sci. 106:3321–3344. doi:10.3168/jds.2022-22454 37028959

[CIT0023] Giannuzzi, D., F. Piccioli-Cappelli, S. Pegolo, V. Bisutti, S. Schiavon, L. Gallo, A. Toscano, P. A. Marsan, L. Cattaneo, E. Trevisi, et al. 2023b. Observational study on the associations between milk yield, composition and coagulation properties with blood biomarkers of health in Holstein cows. J. Dairy Sci 107:1397–1412. doi:10.3168/jds.2023-23546 37690724

[CIT0024] Gianola, D., and D. Sorensen. 2004. Quantitative genetic models for describing simultaneous and recursive relationships between phenotypes. Genetics 167:1407–1424. doi:10.1534/genetics.103.025734 15280252 PMC1470962

[CIT0025] Gross, J., H. A. van Dorland, R. M. Bruckmaier, and F. J. Schwarz. 2011. Performance and metabolic profile of dairy cows during a lactational and deliberately induced negative energy balance with subsequent realimentation. J. Dairy Sci. 94:1820–1830. doi:10.3168/jds.2010-3707 21426971

[CIT0026] Gross, J. J., E. C. Kessler, C. Albrecht, and R. M. Bruckmaier. 2015. Response of the cholesterol metabolism to a negative energy balance in dairy cows depends on the lactational stage. PLoS One. 10:e0121956. doi:10.1371/journal.pone.0121956 26034989 PMC4452704

[CIT0027] Guliński, P. 2021. Ketone bodies – causes and effects of their increased presence in cows’ body fluids: a review. Vet. World. 14:1492–1503. doi:10.14202/vetworld.2021.1492-1503 34316197 PMC8304442

[CIT0028] Hayhurst, C., A. P. F. Flint, P. Løvendahl, J. A. Woolliams, and M. D. Royal. 2009. Genetic variation of metabolite and hormone concentration in UK Holstein-Friesian calves and the genetic relationship with economically important traits. J. Dairy Sci. 92:4001–4007. doi:10.3168/jds.2008-1130 19620683

[CIT0029] Henderson, C. R. 1984. Estimation of variances and covariances under multiple trait models. J. Dairy Sci. 67:1581–1589. doi:10.3168/jds.s0022-0302(84)81480-0

[CIT0030] Heringstad, B., X. L. Wu, and D. Gianola. 2009. Inferring relationships between health and fertility in Norwegian Red cows using recursive models. J. Dairy Sci. 92:1778–1784. doi:10.3168/jds.2008-1535 19307661

[CIT0031] Inoue, K., B. D. Valente, N. Shoji, T. Honda, K. Oyama, and G. J. M. Rosa. 2016. Inferring phenotypic causal structures among meat quality traits and the application of a structural equation model in Japanese Black cattle. J. Anim. Sci. 94:4133–4142. doi:10.2527/jas.2016-0554 27898842

[CIT0032] Jia, Y., and J. L. Jannink. 2012. Multiple-trait genomic selection methods increase genetic value prediction accuracy. Genetics. 192:1513–1522. doi:10.1534/genetics.112.144246 23086217 PMC3512156

[CIT0033] Kato, Y., M. Ito, and H. Hirooka. 2011. Genetic parameters of serum vitamin A and total cholesterol concentrations and the genetic relationships with carcass traits in an F1 cross between Japanese Black sires and Holstein dams. J. Anim. Sci. 89:951–958. doi:10.2527/jas.2010-2872 21148786

[CIT0034] Kessler, E. C., J. J. Gross, R. M. Bruckmaier, and C. Albrecht. 2014. Cholesterol metabolism, transport, and hepatic regulation in dairy cows during transition and early lactation. J. Dairy Sci. 97:5481–5490. doi:10.3168/jds.2014-7926 24952770

[CIT0036] López de Maturana, E., A. Legarra, L. Varona, and E. Ugarte. 2007. Analysis of fertility and dystocia in Holsteins using recursive models to handle censored and categorical data. J. Dairy Sci. 90:2012–2024. doi:10.3168/jds.2005-442 17369243

[CIT0037] Lou, W., H. Zhang, H. Luo, Z. Chen, R. Shi, X. Guo, Y. Zou, L. Liu, L. F. Brito, G. Guo, et al. 2022. Genetic analyses of blood β-hydroxybutyrate predicted from milk infrared spectra and its association with longevity and female reproductive traits in Holstein cattle. J. Dairy Sci. 105:3269–3281. doi:10.3168/jds.2021-20389 35094854

[CIT0038] Lu, Z., Z. Xu, Z. Shen, Y. Tian, and H. Shen. 2019. Dietary energy level promotes rumen microbial protein synthesis by improving the energy productivity of the ruminal microbiome. Front. Microbiol. 10:1–14. doi:10.3389/fmicb.2019.00847 31057531 PMC6479175

[CIT0039] Lucy, M. C., and B. A. Crooker. 2001. Physiological and genetic differences between low and high index dairy cows. BSAP Occas. Publ. 26:223–236. doi:10.1017/s0263967x0003370x

[CIT0040] Martinez-Castillero, M., H. Toledo-Alvarado, S. Pegolo, A. I. Vazquez, G. de los Campos, L. Varona, R. Finocchiaro, G. Bittante, and A. Cecchinato. 2020. Genetic parameters for fertility traits assessed in herds divergent in milk energy output in Holstein-Friesian, Brown Swiss, and Simmental cattle. J. Dairy Sci. 103:11545–11558. doi:10.3168/jds.2020-18934 33222858

[CIT0041] Martinez-Castillero, M., S. Pegolo, C. Sartori, H. Toledo-Alvarado, L. Varona, L. Degano, D. Vicario, R. Finocchiaro, G. Bittante, and A. Cecchinato. 2021. Genetic correlations between fertility traits and milk composition and fatty acids in Holstein-Friesian, Brown Swiss, and Simmental cattle using recursive models. J. Dairy Sci. 104:6832–6846. doi:10.3168/jds.2020-19694 33773778

[CIT0042] Mehtiö, T., P. Mäntysaari, E. Negussie, A. M. Leino, J. Pösö, E. A. Mäntysaari, and M. H. Lidauer. 2020. Genetic correlations between energy status indicator traits and female fertility in primiparous Nordic Red Dairy cattle. Animal. 14:1588–1597. doi:10.1017/S1751731120000439 32167447 PMC7369375

[CIT0043] Mezzetti, M., A. Minuti, F. Piccioli-Cappelli, M. Amadori, M. Bionaz, and E. Trevisi. 2019. The role of altered immune function during the dry period in promoting the development of subclinical ketosis in early lactation. J. Dairy Sci. 102:9241–9258. doi:10.3168/jds.2019-16497 31378488

[CIT0044] Mezzetti, M., L. Cattaneo, M. M. Passamonti, V. Lopreiato, A. Minuti, and E. Trevisi. 2021. The transition period updated: a review of the new insights into the adaptation of dairy cows to the new lactation. Dairy. 2:617–636. doi:10.3390/dairy2040048

[CIT0045] Misztal, I., S. Tsuruta, D. Lourenco, I. Aguilar, A. Legarra, and Z. Vitezica. 2018. Manual for BLUPF90 family of programs. Athens, USA: University of Georgia.

[CIT0046] Mora, M., M. Velasco-Galilea, J. P. Sánchez, Y. Ramayo-Caldas, and M. Piles. 2022. Disentangling the causal relationship between rabbit growth and cecal microbiota through structural equation models. Genet. Sel. Evol. 54:1–14. doi:10.1186/s12711-022-00770-2 36536288 PMC9762025

[CIT0047] Mota, L. F. M., S. Pegolo, V. Bisutti, G. Bittante, and A. Cecchinato. 2020. Genomic analysis of milk protein fractions in Brown Swiss cattle. Animals. 10:1–15. doi:10.3390/ani10020336 PMC7070934 32093277

[CIT0048] Mrode, R. A. , 2014. Linear models for the prediction of animal breeding values. Wallingford, UK: Cabi. doi:10.1079/ 9781780643915.0000

[CIT0049] NASEM. 2021. Nutrient requirements of dairy cattle. 8th rev. ed. Washington, DC: Natil. Acad. Press.

[CIT0050] Ospina, P. A., D. V. Nydam, T. Stokol, and T. R. Overton. 2010. Evaluation of nonesterified fatty acids and beta-hydroxybutyrate in transition dairy cattle in the northeastern United States: critical thresholds for prediction of clinical diseases. J. Dairy Sci. 93:546–554. doi:10.3168/jds.2009-2277 20105526

[CIT0051] Overton, T. R., and M. R. Waldron. 2004. Nutritional management of transition dairy cows: Strategies to optimize metabolic health. J. Dairy Sci. 87:E105–E119. doi:10.3168/jds.s0022-0302(04)70066-1

[CIT0052] Overton, T. R., J. A. A. McArt, and D. V. Nydam. 2017. A 100-year review: metabolic health indicators and management of dairy cattle. J. Dairy Sci. 100:10398–10417. doi:10.3168/jds.2017-13054 29153172

[CIT0053] Pegolo, S., M. Momen, G. Morota, G. J. M. Rosa, D. Gianola, G. Bittante, and A. Cecchinato. 2020. Structural equation modeling for investigating multi-trait genetic architecture of udder health in dairy cattle. Sci. Rep. 10:7751. doi:10.1038/s41598-020-64575-3 32385377 PMC7210309

[CIT0054] Pegolo, S., H. Yu, G. Morota, V. Bisutti, G. J. M. Rosa, G. Bittant, S. Schiavon, G. Bittante, and A. Cecchinato. 2021. Structural equation modeling for unraveling the multivariate genomic architecture of milk proteins in dairy cattle. J. Dairy Sci. 104:5705–5718. doi:10.3168/jds.2020-18321 33663837

[CIT0055] Pegolo, S., D. Giannuzzi, F. Piccioli-Cappelli, L. Cattaneo, M. Gianesella, P. L. Ruegg, E. Trevisi, and A. Cecchinato. 2023. Blood biochemical changes upon subclinical intramammary infection and inflammation in Holstein cattle. J. Dairy Sci. 106:6539–6550. doi:10.3168/jds.2022-23155 37479572

[CIT0056] Premi, M., M. Mezzetti, G. Ferronato, M. Barbato, F. P. Cappelli, A. Minuti, and E. Trevisi. 2021. Changes of plasma analytes reflecting metabolic adaptation to the different stages of the lactation cycle in healthy multiparous Holstein dairy cows raised in high-welfare conditions. Animals. 11:1–18. doi:10.3390/ani11061714 PMC8226749 34201201

[CIT0057] Reynolds, C. K., P. C. Aikman, B. Lupoli, D. J. Humphries, and D. E. Beever. 2003. Splanchnic metabolism of dairy cows during the transition from late gestation through early lactation. J. Dairy Sci. 86:1201–1217. doi:10.3168/jds.S0022-0302(03)73704-7 12741545

[CIT0059] Scutari, M. 2010. Learning Bayesian networks with the bnlearn R package. J. Stat. Soft. 35:1–22. doi:10.18637/jss.v035.i03

[CIT0060] Scutari, M., and R. Nagarajan. 2013. Identifying significant edges in graphical models of molecular networks. Artif. Intell. Med. 57:207–217. doi:10.1016/j.artmed.2012.12.006 23395009 PMC4070079

[CIT0061] Trevisi, E., M. Amadori, S. Cogrossi, E. Razzuoli, and G. Bertoni. 2012. Metabolic stress and inflammatory response in high-yielding, periparturient dairy cows. Res. Vet. Sci. 93:695–704. doi:10.1016/j.rvsc.2011.11.008 22197526

[CIT0063] Valente, B. D., G. J. M. Rosa, D. Gianola, X. Wu, and K. Weigel. 2013. Is structural equation modeling advantageous for the genetic improvement of multiple traits? Genetics. 194:561–572. doi:10.1534/genetics.113.151209 23608193 PMC3697964

[CIT0064] Van den Berg, I., P. N. Ho, M. Haile-Mariam, P. R. Beatson, E. O’Connor, and J. E. Pryce. 2021. Genetic parameters of blood urea nitrogen and milk urea nitrogen concentration in dairy cattle managed in pasture-based production systems of New Zealand and Australia. Anim. Prod. Sci. 61:1801–1810. doi:10.1071/an21049

[CIT0065] Varona, L., and O. González-Recio. 2023. Invited review: recursive models in animal breeding: interpretation, limitations, and extensions. J. Dairy Sci. 106:2198–2212. doi:10.3168/jds.2022-22578 36870846

[CIT0066] Varona, L., D. Sorensen, and R. Thompson. 2007. Analysis of litter size and average litter weight in pigs using a recursive model. Genetics. 177:1791–1799. doi:10.1534/genetics.107.077818 17720909 PMC2147959

[CIT0067] Veerkamp, R. F., B. Beerda, and T. van der Lende. 2003. Effects of genetic selection for milk yield on energy balance, levels of hormones, and metabolites in lactating cattle, and possible links to reduced fertility. Livest. Prod. Sci. 83:257–275. doi:10.1016/s0301-6226(03)00108-8

[CIT0068] Velez, J. C., and S. S. Donkin. 2005. Feed restriction induces pyruvate carboxylase but not phosphoenolpyruvate carboxykinase in dairy cows. J. Dairy Sci. 88:2938–2948. doi:10.3168/jds.S0022-0302(05)72974-X 16027208

[CIT0069] Viturro, E., M. Koenning, A. Kroemer, G. Schlamberger, S. Wiedemann, M. Kaske, and H. H. D. Meyer. 2009. Cholesterol synthesis in the lactating cow: induced expression of candidate genes. J. Steroid Biochem. Mol. Biol. 115:62–67. doi:10.1016/j.jsbmb.2009.02.011 19429461

[CIT0070] Wang, Q., Y. Zeng, X. Zeng, X. Wang, Y. Wang, C. Dai, J. Li, P. Huang, J. Huang, T. Hussain, et al. 2021. Effects of dietary energy levels on rumen fermentation, gastrointestinal tract histology, and bacterial community diversity in fattening male Hu lambs. Front. Microbiol. 12:1–14. doi:10.3389/fmicb.2021.695445 PMC8460862 34566905

[CIT0071] Weber, C., C. Hametner, A. Tuchscherer, B. Losand, E. Kanitz, W. Otten, H. Sauerwein, R. M. Bruckmaier, F. Becker, W. Kanitz, et al. 2013. Hepatic gene expression involved in glucose and lipid metabolism in transition cows: effects of fat mobilization during early lactation in relation to milk performance and metabolic changes. J. Dairy Sci. 96:5670–5681. doi:10.3168/jds.2012-6277 23831100

[CIT0072] Wright, S. 1921. Correlation and causation. J. Agric. Res. 20:557–585.

